# State of the art of real-life concentration monitoring of rifampicin and its implementation contextualized in resource-limited settings: the Tanzanian case

**DOI:** 10.1093/jacamr/dlae182

**Published:** 2024-11-14

**Authors:** Yuan J Petermann, Bibie Said, Annie E Cathignol, Margaretha L Sariko, Yann Thoma, Stellah G Mpagama, Chantal Csajka, Monia Guidi

**Affiliations:** Centre for Research and Innovation in Clinical Pharmaceutical Sciences, Lausanne University Hospital and University of Lausanne, Lausanne, Switzerland; Kibong'oto Infectious Diseases Hospital, Sanya Juu Siha/Kilimanjaro Clinical Research Institute, Kilimanjaro, United Republic of Tanzania; The Nelson Mandela African Institution of Science and Technology, Arusha, United Republic of Tanzania; Centre for Research and Innovation in Clinical Pharmaceutical Sciences, Lausanne University Hospital and University of Lausanne, Lausanne, Switzerland; School of Engineering and Management Vaud, HES-SO University of Applied Sciences and Arts Western Switzerland, 1401 Yverdon-les-Bains, Switzerland; Kilimanjaro Clinical Research Institute Kilimanjaro, Moshi, United Republic of Tanzania; School of Engineering and Management Vaud, HES-SO University of Applied Sciences and Arts Western Switzerland, 1401 Yverdon-les-Bains, Switzerland; Kibong'oto Infectious Diseases Hospital, Sanya Juu Siha/Kilimanjaro Clinical Research Institute, Kilimanjaro, United Republic of Tanzania; Centre for Research and Innovation in Clinical Pharmaceutical Sciences, Lausanne University Hospital and University of Lausanne, Lausanne, Switzerland; Institute of Pharmaceutical Sciences of Western Switzerland, University of Geneva, University of Lausanne, Geneva and Lausanne, Switzerland; School of Pharmaceutical Sciences, University of Geneva, University of Lausanne, Geneva & Lausanne, Switzerland; Centre for Research and Innovation in Clinical Pharmaceutical Sciences, Lausanne University Hospital and University of Lausanne, Lausanne, Switzerland; Institute of Pharmaceutical Sciences of Western Switzerland, University of Geneva, University of Lausanne, Geneva and Lausanne, Switzerland; Service of Clinical Pharmacology, Lausanne University Hospital and University of Lausanne, Lausanne, Switzerland

## Abstract

The unique medical and socio-economic situation in each country affected by TB creates different epidemiological contexts, thus providing exploitable loopholes for the spread of the disease. Country-specific factors such as comorbidities, health insurance, social stigma or the rigidity of the health system complicate the management of TB and the overall outcome of each patient. First-line TB drugs are administered in a standardized manner, regardless of patient characteristics other than weight. This approach does not consider patient-specific conditions such as HIV infection, diabetes mellitus and malnutrition, which can affect the pharmacokinetics of TB drugs, their overall exposure and response to treatment. Therefore, the ‘one-size-fits-all’ approach is suboptimal for dealing with the underlying inter-subject variability in the pharmacokinetics of anti-TB drugs, further complicated by the recent increased dosing regimen of rifampicin strategies, calling for a patient-specific methodology. In this context, therapeutic drug monitoring (TDM), which allows personalized drug dosing based on blood drug concentrations, may be a legitimate solution to address treatment failure. This review focuses on rifampicin, a critical anti-TB drug, and examines its suitability for TDM and the socio-economic factors that may influence the implementation of TDM in clinical practice in resource-limited settings, illustrated by Tanzania, thereby contributing to the advancement of personalized TB treatment.

## Introduction

**Figure 1.**
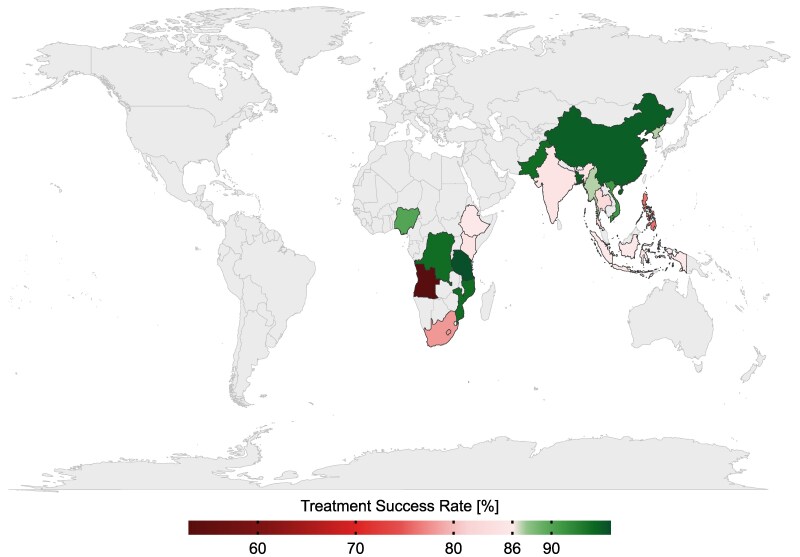
Descriptive map depicting the treatment success rate of all new and relapse cases of TB in countries reporting at least 100 000 TB incidences in 2020, accounting for 82% of worldwide TB incidences.^1^ Ten of 18 countries (including Tanzania), scaled in green, relayed a success rate above the global rate of 86%.^1^ The remaining states, scaled in red, ranged below the global rate.

Despite being curable, TB infection remains one of the leading causes of deaths worldwide, with 1.6 million deaths in 2022.^1^ TB incidences are unequally distributed in the world; 82% of the global TB burden is concentrated in Asia and Africa, with the majority among the low-income countries in 2020 (Figure 1).^1^ Notably, only half of those countries managed to exceed the global treatment success rate of 86% in 2020, and only Tanzania manages to equal the reported clinical success rate of 96% among the screened individuals.^1,2^ In 2015, the WHO reported 430 000 relapse cases of TB accounting for 7% of all TB cases among individuals who had received any prior treatment, suggesting the need for a deeper understanding of the factors influencing treatment outcomes.^3^

Country-specific medical and socio-economic factors create a unique epidemiological context for TB treatment failure, infection rates, morbidity and mortality in each region. For instance, TB disease and management is further complicated by comorbidities, including diabetes mellitus (DM), HIV infection and malnutrition, whose prevalence varies among the TB burden states.^1,4–7^ TB is considered as a marker of HIV positivity due to common TB-HIV coinfection in high HIV prevalence areas, leading to infected individuals’ stigmatization, further impacting their medical care.^8^ Besides, healthcare performance regarding TB varies between states, due to the fluctuation of efficiency of specific features in the cascade of TB care. For instance, whereas most of the high TB burden countries such as Indonesia, China or Tanzania should improve their screening performance, Philippines and Russia are lacking efficient treatment supply services.^9^

From an individual point of view, tackling TB in a standardized fashion based on the ‘one-dose-fits-all’ approach can face limitations because of the known substantial pharmacokinetic (PK) inter- and intra-individual variability displayed by anti-TB drugs, which may lead to higher occurrences of suboptimal plasma concentrations in patients.^6,10^ Furthermore, it has been suggested that low drug plasma concentrations of anti-TB drugs such as pyrazinamide and rifampicin are more likely to happen in patients with the previously mentioned comorbidities.^10–12^

This complex landscape underscores the importance of a comprehensive approach to understand and address TB epidemiology tailored to both the countries’ and the patients’ needs and constraints, to improve treatment outcomes and reduce the global burden of this disease. Therapeutic concentration monitoring, also known as therapeutic drug monitoring (TDM), is one of the most appropriate clinical tools to address the issue by proposing personalized drug dose adjustment based on blood concentration measurements.

Rifampicin is a key component of TB treatment that has gained particular attention due to its critical role in enabling short-course therapy.^13^ This drug is recognized as the most powerful first-line anti-TB drug, and resistance to it is a defining characteristic of MDR-TB.^1^ Indeed, rifampicin is a bactericidal and unique sterilizing agent against *Mycobacterium tuberculosis*. Its mechanism of action relies on the inhibition of bacterial RNA transcription, which subsequently hinders the growth of the bacterium. Due to its efficacy in addressing TB infections, rifampicin has rapidly become a preferred first-line treatment option in the clinical management of TB.^6,14–17^ Whereas substantial variability in exposure has been reported at standard doses, recent interest in a high-dose regimen of rifampicin to shorten treatment duration has revealed even higher drug exposure variability between the patients under increased dosage, underlining interest in concentration monitoring to adjust dosing regimen.^18^

This review aims to provide an overview of the adequacy of rifampicin for TDM including the socio-economic context in low-resource settings, which could possibly hinder widespread implementation of such an approach in clinical practice or worsen treatment outcomes. As this endeavour was conducted in the context of a multidisciplinary and international scientific collaboration for the implementation of Bayesian TDM of rifampicin in Tanzania, the influence of socio-economic and epidemiological factors is illustrated using Tanzania as a case study.^19^

## Methods

Published papers for this review were retrieved in an iterative process through an exhaustive and thorough search of the literature in PubMed, Embase and Google Scholar databases for publications and clinicaltrials.gov for registered trials. The search included ‘rifampicin’, ‘rifampin’, ‘healthcare’, ‘economic’, ‘diabetes mellitus’, ‘malnutrition’, ‘human immunodeficiency virus’, ‘hepatic insufficiency’, ‘tuberculosis’ combined with terms including ‘therapeutic drug monitoring’, ‘pharmacokinetics’, ‘pharmacodynamic’, ‘pharmacology’ and ‘implementation’ and was used to retrieve articles of interest. The latter were scoped and selected based on their relevance to the objective of the review and a cross-reference check was performed to identify missing articles of interest.

## Therapeutic drug monitoring and rifampicin

**Figure 2.**
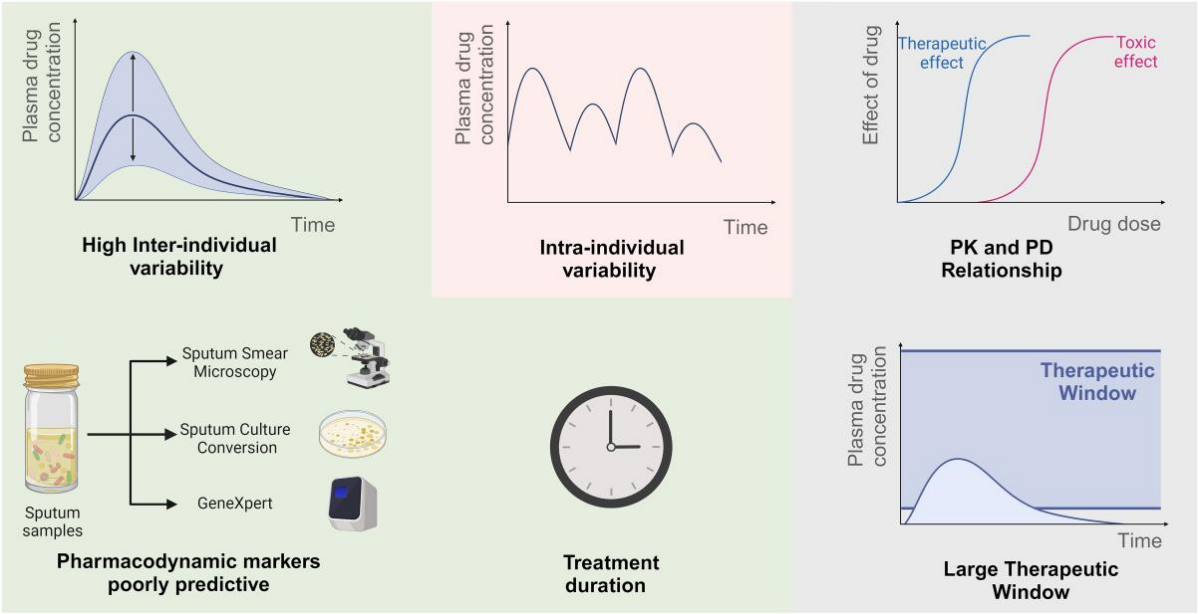
Schematic representation of criteria for drugs suitable for TDM, adapted from Buclin *et al*.^20^ The green-backgrounded criteria—high inter-individual variability, poorly predictive pharmacodynamic markers and long-term treatments—align well with rifampicin's characteristics. In contrast, the red-backgrounded intra-individual variability disfavours use of TDM for rifampicin. Although these aspects of rifampicin are well-established in the literature, the pharmacokinetic-pharmacodynamic (PK/PD) relationship and the resulting therapeutic window (backgrounded in grey) still require further clarification. Created with BioRender.com.

On the verge of the widespread precision medicine era, TDM is a strategy that aligns with this approach by enabling personalized dosage adaptation based on assessment of drug concentration, most commonly in the blood or the plasma.^20^ TDM has been recognized as a beneficial tool in optimizing TB management and has been recommended by major health bodies such as the WHO or the American Thoracic Society in TB drug-resistance guidelines.^10,21–24^ Performing TDM in clinical practice brings major benefits in TB management, including the prevention of toxicity, therapy adjustment for specific patient subsets, identification of potential drug interactions or avoidance of antimicrobial resistance.^10^ Notably, due to its long-term use in treatment, rifampicin meets most criteria of TDM suitability (Figure 2).^20,25–27^

### Rifampicin pharmacokinetic and inter- and intra-individual variability

**Figure 3.**
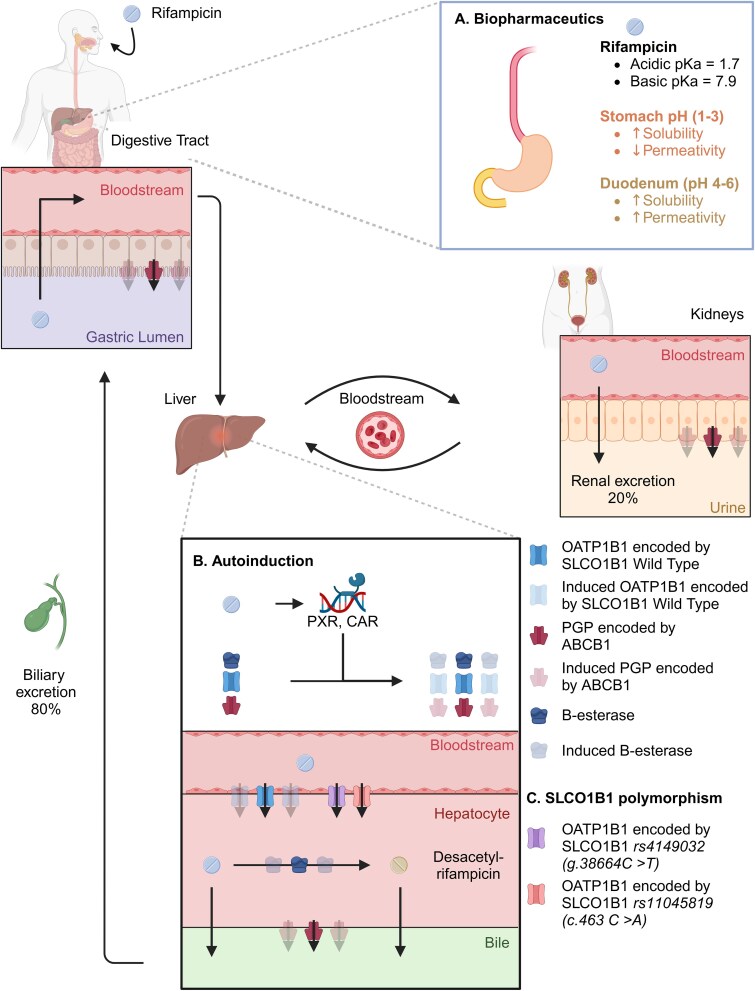
Schematic overview of rifampicin pharmacokinetics and reported parameters influencing rifampicin exposure. (a) Schematic representation of the known biopharmaceutics leading to rifampicin absorption and bioavailability variability. (b) Mechanism of autoinduction occurring after repeated administration of rifampicin due to activation of nuclear receptors pregnane X (PXR) and constitutive androstane receptors (CAR), leading to decreased exposure of rifampicin. This results in enhanced transcription of phase I, II and III metabolic enzymes such as B-esterases and transporters (depicted as transparent), including the solute carrier 1B1 (*SLCO1B1*) and adenosine triphosphate (ATP)-binding cassette B1 (*ABCB1*) genes, encoding respectively the organic anion-transporting polypeptide 1B1 (OATP1B1) transporters and the P-glycoprotein (PGP). (c) SLCO1B1 polymorphisms such as *SLCO1B1 rs4149032* (g.38664C > T) and *SLCO1B1 rs11045819* (c.463C > A) genotypes have been reported to decrease rifampicin exposure. Created with BioRender.com.

Despite its outstanding effect on *Mycobacterium tuberculosis*, rifampicin delivery to the site of action is mediated by complex PK (Figure 3) characterized by extended inter- and intra-individual variability, partly explaining unfavourable treatment outcomes.^11,28,29^

Due to an acidic dissociation constant (p*K*_a_) of 1.7 and a basic p*K*_a_ of 7.9, rifampicin is highly soluble and poorly permeative in gastric physiological conditions (pH 1 to 3) and moderately to highly soluble and highly permeative in the duodenum (pH 4 to 6) (Figure 3a).^30^ The potential 100-fold variation of rifampicin solubility within the stomach depending on its pH impacts the absorption rate and the bioavailability of this drug.^30,31^ Various parameters, including stomach pH, or food and antacid intake but also galenic formulation such as fixed drug combination or single drug formulation are expected to influence rifampicin absorption. Yet, the significance of the impact of those parameters is widely debated.^6,14,30–36^

Rifampicin is primarily metabolized into desacetyl-rifampicin in the liver by hepatic B-esterases and excreted with the bile, and less than 20% of untransformed rifampicin is excreted by the kidneys (Figure 3).^6,13,14,18,36–40^ The significant first-pass metabolism is known to be saturable, due to the hepatocellular intake being mainly driven by the influx transporter organic anion-transporting polypeptide 1B1 (OATP1B1).^6,14,36,37,39^ As rifampicin is also a substrate for the P-glycoprotein (PGP) transporters, absorption, bioavailability and elimination may be influenced by a potential saturation of both metabolizing enzymes and transporters.^36,38^ Saturation of the esterase metabolizing enzymes and efflux and influx channels such as PGP and OATP1B1 is an even more probable consequence of a high-dose regimen exceeding the standard WHO dosing of 10 mg/kg. The widespread adoption of high dosing of rifampicin in clinical practice may result in increased bioavailability and absorption, leading to a non-linear rise in plasma concentrations and extended variability of drug exposure among patients.^14,37,38,41–43^

After repeated administration, rifampicin induces its own elimination and metabolism reducing its plasma concentration, a process referred to as autoinduction (see Figure 3b).^6,42,44^ The latter is notably mediated by its binding and activation of the nuclear receptor pregnane X (PXR) and constitutive androstane receptor (CAR).^37,45–47^ This leads to an increased transcription of genes responsible for phase I, II and III metabolic enzymes including hepatic B-esterases and transporters, namely the solute carrier organic anion transporter family member 1B1 (*SLCO1B1)* and ATP-binding cassette B1 (*ABCB1*) genes encoding, respectively, the OATP1B1 transporters and the PGP.^40,45,46^ The up-regulation of such enzymes and transporters results in an increased clearance, thus decreasing exposure and half-life of rifampicin over time. As a consequence, the usual half-life of 3–4 h of rifampicin after single doses in patients with normal liver function drops to 1–2 h after several administrations.^10,36^ The induction steady state is usually achieved after 1 or 2 weeks of rifampicin continuous intake, and return of baseline enzyme function is reached after 2–4 weeks of rifampicin treatment discontinuation.^6,10,37,40,42,44,48,49^

As illustrated above, several parameters including formulation or comorbidities can influence rifampicin PK profile, thus leading to substantial inter- and intra-individual variability in drug concentration and effect.^14^ Despite the extensive and numerous rifampicin PK studies published, the extent to which those factors influence rifampicin PK remains highly variable and is difficult to predict, preventing accurate *a priori* dosage adjustment.^49^ Potential wide implementation of a high-dose regimen would suggest additional careful monitoring as a consequence of non-linear and saturable PK.^18,44^ For instance, Sturkenboom *et al*. reported that rifampicin doses of 9.6 mg/kg and 20.5 mg/kg (approximately 2-fold increase) resulted in an AUC_24h_ of 2.3 and 130 mg·h/L (approximately 56-fold increase), respectively, confirming the difficulty in predicting rifampicin exposure based on the dose.^50^

Whereas inter-individual variability advocates for TDM, intra-individual variability, notably described by autoinduction or variation of stomach pH, reflects an unpredictable PK profile from one occasion to another, constituting a drawback for TDM use in rifampicin.^20^ Currently, appropriate prediction of variation of rifampicin PK based on patient characteristics or drug formulation is still lacking.^48,49^ Phenotypic approaches such as TDM allow a better picture of the drug’s PK in the individual, thus limiting treatment failure by providing tailored drug adjustment.

### Absence of a pharmacodynamic marker

New biomarkers for assessment of TB treatments are being intensively studied. Still, the best characterized biomarker of treatment outcome remains the sputum culture conversion.^51,52^ However, culture-based biomarkers, such as smear microscopy and sputum culture, are poorly predictive of treatment outcomes, as they have a low sensitivity and a modest specificity for prediction of treatment relapse or failure.^52–54^ Moreover, these methods can take weeks before returning results and possibly fail to depict treatment failure or resistance at appropriate timings.^27,52,55^ The newly established GeneXpert test is delivering results faster but still has limitations, such as failure to distinguish living and non-living *Mycobacterium tuberculosis* and a high cost, hampering its deployment in high TB burden countries often associated with poor resources.^52,55^

### Pharmacokinetic targets and pharmacokinetic-pharmacodynamic relationship

Since the 1990s, the plasma peak concentration (*C*_max_), ranging from 8 to 24 mg/L, has been employed as an efficacy target for rifampicin TDM.^56,57^ The proposed TDM sampling times of 2 and 6 h post dose enable capturing *C*_max_ under normal or delayed absorption.^6,10,48,49,58^

Using *C*_max_ as a TDM target is rather debatable as it represents an expected concentration range after a standard dosage regimen based on observed PK parameters, without any link to efficacy targets.^49,56,57,59^ Whereas previous studies linked slow treatment responses with low *C*_max_,^6,27,60^ recent systematic reviews and meta-analyses have failed to show clear evidence of a relationship between peak concentration and treatment outcomes.^53,58^ The systematic review and meta-analysis of Perumal *et al*. based on 20 articles and 2109 patients showed that rifampicin below the traditional *C*_max_ range slightly but non-significantly increased the risk of poor outcome, with a relative risk of 1.40 (95% CI, 0.91 to 2.16).^53^ Two-thirds of the reported rifampicin concentrations 2 h post-dose were below target in the review of Mota *et al*. pooling 41 studies and 2727 patients, but no clear association between low peak concentrations and unsuccessful outcomes was identified.^58^ In addition, the *C*_max_ measure as an efficacy target is further compromised by the known significant variability in the rifampicin absorption phase.^6^

Over the past two decades, growing evidence supports the AUC_24h_/MIC ratio as a more suitable therapeutic target.^6,11,27,48,53^ The *in vivo* study conducted by Jayaram *et al*. suggested a minimal threshold for efficacy of AUC_24h_/MIC at 271 h.^48,49,61^ This therapeutic target is advocated by a panel of 51 experts, who recommend its application under the supervision of a multi-professional team experienced in TDM.^11^ The same authors also stipulate the careful use of their reported MIC, due to the systematic methodological differences to quantify it. The clinical trial conducted by Zheng *et al*. on 168 patients in China determined a therapeutic target of AUC_24h_/MIC between 435 and 683 h, based on 79% and 97% of successful treatment outcomes related to AUC_24h_/MIC lower and higher than 435 h, respectively.^28^

Although the concentration-toxicity relationship still needs to be clarified, some studies suggest that the exposure is an appropriate predictor of adverse events occurrence.^18,28^ The study of Zheng *et al*. included patients treated by a standard combination therapy of rifampicin 450 and 600 mg for patients weighing less and more than 50 kg, respectively, in combination with standard doses of isoniazid, pyrazinamide and ethambutol.^28^ The risks of acute kidney injury and drug-induced liver injury were significantly increased for rifampicin AUC_24h_ exceeding 82 mg·h/L or an AUC_24h_/MIC over 683 h.^28^ Within that dose range, the patients displayed an AUC_24h_ varying between 33 and 127 mg·h/L, with 11% of patients above the 82 mg·h/L toxicity threshold.^28^ Similar to Zheng *et al.*, Te Brake *et al*. in their high-dose rifampicin studies also reported that AUC_24h_ strongly predicted adverse events together with a considerable variability in drug exposure, but did not recommend a maximal safety threshold.^18,28^ In the 40 mg/kg arm, considered by Te Brake *et al*. as the maximum tolerated dose, the average AUC_24h_ after single and combination therapies was 387 mg·h/L (range: 201–847 mg·h/L), and 257 mg·h/L (range: 173–349 mg·h/L), well above the previously mentioned toxicity thresholds.^18^ The unclear concentration-toxicity thresholds together with the substantial inter-individual variability in rifampicin exposure emphasize the need for evaluation of drug dosage on a case-by-case safety evaluation.

AUC_24h_ or AUC_24h_/MIC remains impractical, expensive and laborious, especially when relying on full PK sampling for calculation, and is therefore rarely used in clinical settings. However, more modern approaches using model-based strategies have been increasingly recognized for their ability to predict patients’ PK profiles and relevant parameters. For instance, limited sampling strategies employing population PK models offer an appealing and efficient alternative, using three strategically timed samples to accurately predict AUCs.^62,63^ Similarly, future model-based strategies could better estimate patients’ *C*_max_ than the standard 2 and 6 h post-dose sampling.^48,63^

In conclusion, the large variability in rifampicin dose–concentration, in drug exposure together with some evidence of concentration-effect and toxicity relationships favours rifampicin TDM, though the toxicity range for rifampicin has still not been clearly established and remains arbitrary. Efficacy targets based on MIC can constitute a hurdle, as individual determination of the MIC is not conducted in routine clinical care and appropriate choice can be difficult, as various MICs have been reported in the literature.^28,64–67^ A proper definition of the therapeutic window for rifampicin still needs further investigations to consolidate TDM practices for rifampicin.

## Tuberculosis and epidemiological situation in Tanzania

### Tuberculosis and diabetes mellitus comorbidities

Tanzania suffers from a high burden of infectious and communicable diseases, and non-communicable disease prevalence rises notably due to the spread of a Western lifestyle and increased life expectancy.^5^ For instance, the incidence of patients diagnosed with DM and TB ranges from 4% to 17% in rural and urban settings, respectively.^5^ Although DM constitutes a risk factor for TB infection,^11,36^ patients suffering from both TB and DM are more likely to experience treatment failure or relapse.^5,6,11^ Physiological alterations, such as delayed gastric emptying associated with uncontrolled DM, may influence rifampicin absorption.^5,6,11,14,36^ Yet, the recent systematic review by Muda *et al*. failed to confirm the impact of DM on rifampicin PK, since only one of five population PK studies found a significant PK alteration in TB-DM patients.^14,68^ As opposed to a decreased and delayed absorption rate expected due to the hyperglycaemic condition, this article reported an increased absorption rate in patients with TB-DM, emphasizing the controversial nature of the found effect.^14,68^ In non-compartmental studies, no significant effect on rifampicin PK associated with DM was reported by Ruslami *et al.*, whereas others showed a rifampicin exposure reduced by half in patients diagnosed with both DM and TB compared with TB alone.^69–71^ Some evidence to date suggests that some DM patients infected with TB are potentially underexposed, leading to a risk of treatment failure as well as acquired drug resistance. However, the impact of DM on rifampicin PK remains inconclusive.^14^

### Tuberculosis and human immunodeficiency virus coinfections

Because HIV targets the gut-associated lymphoid tissues, the resulting enteropathy is known to induce malabsorption and reduce TB drug availability.^6,10,72–75^ Yet, evidence is still conflicting as studies, including a meta-regression analysis, did not find any association of lower rifampicin exposure with HIV-positive patients.^37,49,76–78^ In this population, a careful check and management of HIV drug and co-medication interaction is recommended due to rifampicin induction. This is especially true in sub-Saharan African regions, as, for instance, TB-HIV coinfection affected up to 20%–49% of the TB Tanzanian population in 2017.^1,4^ For the treatment of HIV infection, the WHO recommends dolutegravir as the first-line antiretroviral drug regimen.^79^ As dolutegravir is metabolized primarily by 5′-diphospho-glucuronosyltransferase 1A1 (UGT1A1) and secondarily by cytochrome P450 3A4 (CYP3A4), a major drug interaction with rifampicin-based therapies is expected due to the induction of those enzymes. This has led to the current WHO dosing recommendation of 50 mg dolutegravir twice daily instead of the standard 50 mg once daily for people with TB and HIV coinfection.^79,80^

### Tuberculosis and malnutrition comorbidities

The scope for malnutrition definition is broad, and it includes the lack of protein and/or calorie intake or a BMI less than 18.5 kg/m^2^.^6,7,81^ Malnutrition can impact the individual by weakening the immune system and, with a prevalence of 22% in Tanzania, it is the number one risk factor for TB infection.^81,82^ Malnourished patients are also more likely to suffer from greater TB severity and decreased TB treatment success.^81^ The effect of poor nutrition on rifampicin PK is not clearly established, as it can increase, decrease or have no significant impact on drug concentrations.^6,7^ Gastrointestinal inflammation resulting from malnutrition can impair the absorption and bioavailability of rifampicin by altering the pH or delaying the gastric emptying.^7,83^ Malnutrition can lead to lower production of plasma proteins, such as albumin, potentially impacting the distribution and the hepatic elimination of rifampicin by modifying its free fraction.^7^ Due to the high affinity of the latter on plasma proteins (80%–90%), a rise of the free fraction is expected, potentially impacting the distribution and the hepatic elimination of rifampicin.^6,7,36,84^ Although the subsequent increase of the free fraction of rifampicin can enhance the drug's availability at the action site,^6,83^ a systematic review reported enhanced hepatic elimination for albumin-bound protein such as rifampicin.^7^ Increased free fraction results in greater availability of rifampicin for hepatic uptake, resulting in augmented metabolism and elimination.^7^

### Gene polymorphism in Tanzania

The magnitude of the influence of gene polymorphisms on the overall exposure of rifampicin is debated and has been extensively reviewed by Thomas *et al.* and assessed in the systematic review led by Muda *et al*.^14,46^ Among SNPs of importance in the African sub-Saharan population, the *SLCO1B1* genotypes encoding for OATP1B1, including *rs4149032* (g.38664C > T) and *rs11045819* (c.463C > A), stand out due to their predominance in Black Africans.^6,85,86^ Lower rifampicin exposure associated with those genotypes has been described in the literature.^46,75,85^ In a study including 361 Tanzanians, *SLCO1B1 rs4149032* and *rs11045819* polymorphisms were reported to be carried by 92.3% and 9.4%, respectively, of the population, increasing the risk of lower rifampicin exposure.^87^ Yet, other studies found no significant genetic influences in other African or Asian countries.^14,47,72,88^ Likewise, no significant genetic effects on rifampicin exposure have been depicted by the review of Thomas *et al*. and the systematic review of Muda *et al*., indicating a lack of evidence associating genetic variants and rifampicin plasma concentration variations.^14,46^

## Socio-economic status in Tanzania

Worldwide, the economic impact of TB on households is significant.^89^ As reported by the WHO, approximately half of TB patients face catastrophic expenditures with costs generated by the disease exceeding 20% of household income.^1^ Despite TB treatment being provided free of charge in most high TB burden countries, patients often encounter considerable financial strain. The latter is due to indirect costs like transportation, nutritional supplements and lost income from time spent seeking care or hospitalization, or direct costs such as follow-up visits or radiography.^89^ These financial issues disproportionately impact the poor, exacerbating their financial hardship and/or discouraging them from seeking treatment, which explains at least partially why only one-third of the Tanzanian ill population seek medical services.^89,90^

Financial support by universal health coverage is still lagging in Tanzania and fails to ensure healthcare with accessible services at lower costs and minimal drawbacks even for infectious diseases of poverty.^91^ The introduction by the government of various social healthcare policies led to the deployment of national health insurance, to which only 32% of the Tanzanian population subscribed in 2019.^91^ Although more affordable, national health insurance still provides limited financial coverage and results in out-of-pocket expenditures relying on patients’ financial capacity.^90^

Furthermore, the current Tanzanian health system operates in disease-specific programmes, with less emphasis on the influences that comorbidities and their treatments have on each another, limiting healthcare delivery and intervention. This vertical compartmented approach of the Tanzanian health system results in delayed care for dual or more diseased individuals, showing overall inefficiency and impaired cost-effectiveness.^5^

Such a systemic, social, economic and epidemiological situation in Tanzania contributes to TB transmission and hinders treatment adherence and favourable patient outcomes, impeding efforts to eliminate TB. This context highlights the need to reduce the financial burden associated with healthcare costs for patients and their families, as well as developing integrative health programmes centred on patient specificity and comorbidities.^5,89^ As such, TDM is an appealing solution for meeting the needs of individuals, but is not without obstacles that need to be considered for proper implementation.

## Current hurdles and plausible solutions for the implementation of therapeutic drug monitoring in tuberculosis management

**Figure 4.**
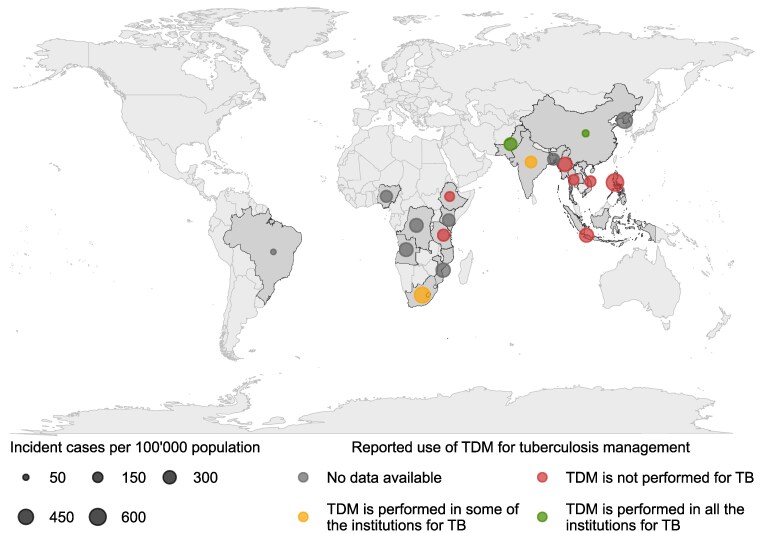
Descriptive map displaying the clinical use of TDM for TB according to Margineanu *et al.* and the incidence of TB in the top highest-burden TB countries in 2021, retrieved from the WHO.^1,92^ Countries reporting at least 100 000 TB incidences and accounting for 84% of worldwide TB incidences in 2021 are displayed according to the circle sizes from highest to lowest incidence of TB per 100 ' 000 population.^1^ TDM for TB management per country described by colour is based on the reported participants’ claims of TDM use in their institutions according to the survey of Margineanu *et al.*^92^ Among the 19 TB burden countries shown on the map, 8 reported absence of TDM use in their reported institutions, 2 practised TDM in some of their institutions and 2 performed TDM for TB management. Further investigations are required to better assess TDM use in TB, as no information was available in seven of the highest TB burden countries.

A recent international survey of the use of TDM for TB management in 86 subjects spread over 46 countries revealed that TDM was performed in approximately half of the survey participants’ institutions.^92^ Strikingly, participants from high burden TB countries such as Philippines, Indonesia or African sub-Saharan countries, including Tanzania, reported the absence of TDM in clinical practice (Figure 4).^1^ Several factors can hinder implementation of TDM in endemic countries, as further illustrated in Table 1.

**Table 1.** Summary of challenges and their solutions for TDM implementation in low-resource settings

Category	Challenges	Proposed solutions
Access to laboratory	Logistical hurdles, including poor sample collection infrastructure and cold-chain requirements	Development and implementation of point-of-care and fast screening assays that do not require complex logistics Promotion of dried blood spot assays, which are more feasible in resource-limited settings
Analytical methods	High costs associated with HPLC and LC-MS/MS technologies for drug quantification	Development and implementation of less expensive technologies such as point-of-care and fast screening assays
Knowledge on TDM	Lack of widespread information and awareness concerning the benefits and implementation of TDM	Provision of comprehensive training for local healthcare professionals on TDM practices and interpretation
	Absence of clear and consistent guidelines for TDM	Establishment and dissemination of clear clinical guidelines for TDM in TB management, tailored to local contexts
	Lack of TDM experts and insufficient training for healthcare providers on the interpretation and application of TDM data	Encouragement of mentorship programmes and e-learning platforms to build local expertise in TDM Implementation of clinical decision support systems or MIPD to assist clinicians in making informed dosing adjustments

### Knowledge gap and therapeutic drug monitoring

The survey reported poor widespread information about TDM use and its benefits, with 37% of the participants claiming that a knowledge gap within the medical staff is hindering its implementation, and 35% stating that TDM use was prevented by insufficient funding and guideline usage.^92^ As described earlier, the complex PK of rifampicin calls for experts in TDM to propose appropriate dosing adjustment, and these are often lacking in resource-limited countries.^92^ Furthermore, in the absence of a clear consensus for an appropriate therapeutic target of rifampicin, guidelines do not provide well-defined pharmacokinetic/pharmacodynamic (PK/PD) ranges, and the AUC_24h_/MIC targets bring logistical and economic hurdles.^24^ This situation impairs the programmatic establishment of TDM, hindering its widespread use in various healthcare facilities.

Model-informed precision dosing (MIPD), relying on specific population PK models and a Bayesian approach, allows for a dosing adjustment tailored to the patient characteristics. Such a medical tool can be further improved by a clinical decision support system, facilitating the decision process of clinicians regardless of the in-depth knowledge of TDM experts.^93,94^ Subsequent training of health professionals using e-learning, mentorship or workshops, as underlined by Mpagama et *al.*, could facilitate the integration of TDM in the healthcare system.^5^

### Limited laboratory resources

Access to a suitable laboratory and its expensive equipment can constitute a drawback to the deployment of TDM, especially in resource-lacking settings. Highly sensitive and selective LC-MS/MS technology offers high-throughput capacity but can be quite costly and demanding, as regards the training of highly qualified staff and need for reliable infrastructures and a stable power supply. HPLC coupled with UV detectors represents an appropriate and less expensive alternative, although such methods demand extensive sample preparation and longer run times.^10,25,95,96^

As stated before, patients can be reluctant to attend clinics due to indirect out-of-pocket expenditures incurred by travelling or missed working day(s).^89^ Thus, development of fast and cheap semi-quantitative point-of-care tests easily accessible by the population would enable screening of under- or overexposed patients at the community level. Likewise, development of analytical methods not relying on a cold chain for sample storage, such as dried blood spot sampling, benefits the logistics in the Tanzanian climate.^97^ Selected out-of-range patients can be referred to the regional level to confirm suspicion with LC-MS/MS or HPLC/UV, hence prioritizing and diminishing the cost of TDM.^10,12^ Processing patients’ samples also generates costs, notably due to the laboratory work or patients’ travel expenses. Limited sampling strategies represent legitimate solutions to decrease the number of samples required to monitor drug concentrations of patients and reduce the overall financial cost of TDM.^62,63,98,99^

### Cost-effectiveness of therapeutic drug monitoring

Interestingly, almost half of the participants from the mentioned survey considered TDM to be cost-effective, with only a minority (∼10%) stating the opposite.^92^ Although the cost-effectiveness of TDM has been demonstrated in other fields,^100–102^ data demonstrating cost-effectiveness of TDM for anti-TB drugs are still insufficient, and the percentage of patients whose treatment would be shortened with TDM needs to be established.^2^ A standard first-line anti-TB treatment lasts for 6 months and displays a 96% success rate in clinical settings; yet it has been shown that treatment duration can be closer to 12–18 months with success rates in real life settings nearer to 75%–80%.^2^

Poor patient outcomes, such as treatment failure, relapse and/or acquired drug resistance, fail to contain TB and increase its spread, thus generating costs.^60^ Such outcomes have been associated with low drug exposures, which could be assessed and averted with TDM.^2^ Although generalized TDM for every TB patient seems unrealistic, optimized prioritization of its use could benefit the management of the TB epidemic.^12^ Henceforth, HIV, DM, gastrointestinal disorders, malnutrition, high rifampicin dosage, and absent or slow treatment response stand for cases in which TDM could be recommended.^6,10,48^

Overall, funding constitutes one of the major hurdles for the implementation of TDM. It impacts staff education on TDM, and the availability of drug monitoring regardless of patient economic status or access to costly analytical apparatus for measuring blood concentrations.

## Therapeutic drug monitoring in Tanzania

Given these challenges, the implementation of TDM in low-resource settings like Tanzania might seem unrealistic.^103^ However, many of these technical obstacles can be addressed through the adoption of appropriate technologies, such as point-of-care testing, MIPD or dried blood spot techniques, as illustrated before. Despite these solutions, the limited adoption of TDM in high-burden TB countries may reflect insufficient interest or awareness of TDM as a therapeutic strategy, further exacerbated by the lack of local clinical expertise, such as clinical pharmacologists and pharmacists. Contrary to this perception, significant efforts have been made to publish implementation strategies^97,104,105^ and establish clinical standards for TDM in TB management.^11^ These developments address the hurdle of a lack of guidelines and provide a framework for integrating TDM into TB programmes, thus rooting its adoption deep in the healthcare system.

Although deprived of TDM, Tanzania, like many other resource-limited countries, has committed to the END-TB by 2035 initiative by embracing innovations and new technologies.^106^ The country uses the Kibong’oto Infectious Diseases Hospital, a national reference facility for TB management with a state-of-the-art laboratory, to introduce and adapt innovative technologies aimed at optimizing clinical management of TB. The facility is actively involved in TB research, developing analytical methods with pragmatic applications. For instance, the facility has developed analytical methods for anti-TB drugs in more convenient matrices than plasma, such as dried blood spot sampling, to address cold-chain logistics challenges in Tanzania’s climate, while also exploring saliva-based point-of-care testing using mobile UV spectrometers for personalized dosing strategies.^97,107^ To bridge the gap of TDM knowledge caused by the lack of experts, the same facility is involved in the development of an MIPD to support the implementation of TDM in the country and educate healthcare professionals in the process.^19^ The growing interest in TDM is further evidenced by its incorporation into large-scale initiatives, such as the Adaptive Diseases Control Expert Program in Tanzania. This protocol acts on a systemic level, offering pragmatic solutions to clinically relevant and tangible issues, as well as financing implementation of impactful measures such as TDM and requisite resources.^5^

## Conclusion

In low-resource settings, where socio-economic disparities are prevalent and TB remains a substantial health burden, the pursuit of effective TB treatment has encountered multifaceted challenges. The PK of rifampicin, a cornerstone of TB therapy, is marked by substantial inter- and intra-individual variability, potentially further complicated by the prevalence of comorbidities such as HIV, DM and malnutrition. The extent to which rifampicin exposure varies depending on known parameters, such as comorbidities, pharmacogenetics and formulation, is still debated, advocating for the use of a concentration-based approach for dose adjustments.

The socio-economic context of low-resources settings, as illustrated in Tanzania, presents notable challenges to healthcare delivery, with high healthcare costs imposing a substantial burden on patients and their families. This situation may lead to compromised treatment adherence and less favourable patient outcomes. Implementing integrated healthcare programmes that prioritize patient-specific care and alleviate financial barriers is crucial for effectively addressing TB. Precision medicine tools such as TDM constitute legitimate solutions to investigate in low-resource settings such as the Tanzanian context.

Evidence supporting TDM for improving TB treatment outcome based on rifampicin is still lacking, and concentration monitoring needs further investigations to demonstrate its concrete benefit. Hurdles to the implementation of TDM in resource-limited settings are many but are highly impacted by the lack of funding. Limited access to suitable laboratories and expensive analytical equipment, and the shortage of trained staff constitute major impediments preventing large-scale adoption of TDM. Addressing those challenges and expanding the scope of TDM are crucial steps in improving TB treatment outcomes and working towards the goal of TB eradication.
